# Hare-Lip and Cleft Palate

**Published:** 1913-03

**Authors:** 


**Hare-lip and Cleft Palate.** By James Berry, B.Sc. (Lond.), F.R.C.S., and T. Percy Legg, M.S. (Lond.), F.R.C.S. Pp. xvii, 324. London: J. and A. Churchill. 1912. 12s. 6d. net.

This is perhaps the most satisfying work on the above subject we have read, and it will well repay perusal by all who take an interest in the repair of such deformities.

The preliminary chapters on development and anatomy contain just as much information as is necessary for the proper appreciation of the various types of deformity that occur, and the methods of dealing with them. We think the chapter on the treatment of hare-lip would have been more complete had there been included an account of some of the better known incision-plans in addition to those recommended by the authors.

The importance of invariably preserving the pre-maxilla is most properly emphasised.

No definite pronouncement is made as to the best age at which a cleft palate should be closed, but we gather from the statistics given that the authors are not in favour of very early operation, and the expert opinion that speech-training is best commenced at the age of six years seems to support this view.

The preliminary routine removal of the mass of adenoids which is so frequently present is deprecated, for the reason that it helps to block the naso-pharynx and so assists in correct articulation ; but we doubt whether this advantage outweighs the many disadvantages of the presence of such a mass.

Langenbeck's operation, as practised by the authors, is described in full detail, and will be of the utmost service to beginners in this operation.

Amongst other operations mentioned that of Brophy receives full attention. Its dangers and high mortality are thoroughly discussed, and lead to the opinion that it is questionable whether it is desirable to do an operation which undoubtedly involves considerable risk when the same result may be obtained by waiting for a year or two.

A useful addition to the book are chapters on secondary operations on the palate and lips, from which many practical hints may be obtained.

